# Priority-Setting and Values: A Qualitative Study of the Danish Medicines Council

**DOI:** 10.1007/s11673-025-10470-3

**Published:** 2025-10-01

**Authors:** Anna Christine Dorf, Andreas Albertsen, Lasse Nielsen

**Affiliations:** 1 Political Science, Aarhus University, Bartholins Allé 7, 8000 Aarhus C, Denmark; 2 Centre for the Experimental-Philosophical Study of Discrimination, Political Science, Aarhus University, Bartholins Allé 7, 8000 Aarhus C, Denmark; 3 Philosophy, Department of Design, Media and Educational Science, University of Southern Denmark, Campusvej 55, 5230 Odense M, Denmark

**Keywords:** Prioriity-setting, Discrimination, Health, Cost-effectiveness, Priority setting, Bioethics

## Abstract

As priority setting committees become commonplace in contemporary welfare states, it becomes increasingly important to understand how they operate. This article contributes to our understanding of contemporary priority setting by examining how the Danish Medicines Council (DMC) makes and justifies its decisions, as well as the role of different (and perhaps conflicting) concerns and values in these decisions. We conducted seventeen interviews with DMC members and observed three DMC meetings spanning five days. Firstly, we find that health-related effect is the most crucial factor in DMC members’ recommendations of newly proposed medicines and that discussions of effects take precedence over other considerations in council deliberations. Secondly, we find that the ability of DMC members to adequately assess the effect of newly proposed medicines is often significantly limited by poor data quality and a lack of sufficient documentation, which shifts the DMC’s task from making recommendations on an informed basis to providing estimated assessments of the expected effect. In these circumstances of uncertainty about effect, recommendations are influenced by considerations such as the age of patients and the rarity of the disease. This raises significant moral issues in which the DMC has no particular expertise.

**Supplementary Information:**

The online version contains supplementary material available at 10.1007/s11673-025-10470-3.

## Introduction

Around the world, the combination of tight budgets and the emergence of expensive new medicines and treatments puts societies in a difficult position. Tough choices must be made when the demand for treatment far outstrips the resources societies have allocated for healthcare spending (Bognar and Hirose 2022; Ubel 2000). Therefore, the question of what values or principles to apply and how decisions should be institutionalized are pertinent.

There is considerable heterogeneity in priority setting (Seixas 2018). It can range from informal, non-transparent, ad hoc priority setting at the bedside to Committees or public institutions deciding which treatments or medicines to employ in the healthcare system. A famous example of this type of priority setting is the public consultation conducted by the Oregon Commission in 1989 (Dixon and Welch 1991; Hadorn 1991).

However, efforts to introduce priority setting institutions go much further. An important aspect of this has been the introduction of cost-effectiveness analysis into priority setting, which has become increasingly common in Western welfare state schemes for healthcare priority setting. The National Institute for Health and Clinical Excellence (NICE) was established in the United Kingdom in 1999 as one of the first institutes to provide official guidance on healthcare priority setting and resource allocation (Charlton 2020, 2022). Since then, various instantiations of similar institutions have spread across Europe and the rest of the world. Prominent examples include New Zealand (Cumming 1994), Norway (Wisløff 2015), Sweden (Drees et al. 2021; Garpenby and Bäckman 2016), Israel (Shani et al. 2000), the Netherlands (Honigsbaum et al. 2018), Canada (Husereau et al. 2010), the United States, and Spain (Sabik and Lie 2008; Noorani et al. 2007). In 2017, the Danish regions formed the Danish Medicines Council (DMC) to establish a rapid process for evaluating new medicines and to ensure that new medicines approved for use are proven effective for patients (Danske Regioner 2017). This followed a lengthy debate on the merits of explicit priority setting (Det Etiske Råd 1996; Pedersen 2021, 2015).

The question of which values to use in priority setting is the subject of a significant philosophical debate concerning, for example, the permissibility of cost-effectiveness as a priority setting criterion (Bognar and Hirose 2022; Broome 1993; Harris 1987; Nord et al. 2009) and its alleged tendency to discriminate against vulnerable groups such as people with disabilities (Albertsen et al. 2025; Hadorn 1992; Harris 2005; Gold et al. 2002; Dolan et al. 2005; Hadorn 1991) or senior citizens (Brock 1989, 2004, 2009; Harris 1985, 1987; Tsuchiya 2000; Farrelly 2008; John et al. 2017; Albertsen 2023).

Given the number of actual priority setting institutions, different countries have elaborated (with varying degrees of detail) the principles that should guide such priority setting. In Norway, the first Lønning Committee (from 1987) proposed principles of severity and efficacy for healthcare priority setting, and Lønning II added cost-effectiveness (Wisløff 2015). The Dunning report was presented to the Dutch Cabinet in 1991 in the Netherlands. It concluded that health services within the basic healthcare package should satisfy four criteria: necessary care, effectiveness, efficiency, and individual responsibility (Hermans and Exter 1998). In Sweden, the Parliament established the Parliamentary Priorities Commission in 1992 and later, in 1997, amended the Health and Medical Services Act following the recommendations of the Commission. This established three criteria for priority setting (in decreasing order of importance): the principle of human dignity, the principle of need and solidarity, and the principle of cost-effectiveness (Drees et al. 2021). In New Zealand, the National Health Committee has identified four values: benefit (effectiveness), fairness (equity), consistency with community values, and efficiency (The National Health Committee 2002). However, as Charlton et al. point out, it is essential to distinguish between formulated values at the most abstract level and case-based judgements at the applied level, and as such, understanding value-based recommendations for priority setting requires careful attention to the chain of reasoning underlying the process of specification from the former to the latter (Charlton et al. 2024). In this respect, the present study adds to existing studies of the DMC based on stakeholder interviews (Bladt et al. 2023), and analysis of the DMC's recommendations and assessments (Petersen et al. 2023; Rasmussen et al. 2022).

Essential aspects of priority setting cannot be identified by analysing principles or decision-making processes. Decisions are often made through debate and deliberation in committees that apply the principles. Their task is to use the principles and to understand this application (e.g., how the principles work in real life), and we need to analyse not only their decisions but also the preceding discussions. This is important because there may be a divergence between principle and practice. Indeed, previous studies have shown such a divergence. In Norway, a study showed that although the official principles were often used, they were unsystematic and inconsistent (Wester and Bringedal 2018). But it is also essential because even without outright inconsistencies, as the examples above illustrate, the values formulated to guide these institutions are typically broad and open to interpretation, leaving much unresolved about how they must be balanced in case of conflict. We must learn from those undertaking the task to properly understand this balancing.

To contribute to our understanding of contemporary priority setting, the article examines how the DMC makes and justifies decisions and the role of different (and perhaps conflicting) concerns and values in these decisions. Our contribution consists of two main findings. First, the health-related effect is the most crucial factor in DMC members’ recommendations. Second, the ability of DMC members to adequately assess the effect of newly proposed medicines is often limited by poor data quality. Here, the DMC’s recommendations are influenced by several other considerations, such as the age of patients and the rarity of the disease, which raise important moral issues in which the DMC has no particular expertise.

The importance ascribed to effect by DMC members and the difficulty of assessing effect is interesting and surprising for several reasons. First, it portrays the role of the DMC differently than many available public descriptions. Here, the focus is very much on cost-effectiveness, and the task is often portrayed as deciding whether a known effect is worth the price. A second reason why our findings are interesting is that the philosophical debate over priority setting shares this affinity for placing cost-effectiveness at the centre of the discussion without adequately acknowledging the uncertainty about effects that influence real-world priority setting decisions.

## The Case of the DMC

The DMC was established in 2017 (Danske Regioner 2017). It consists of three bodies: special committees related to specific medical fields,^1^ a secretariat, and the Council. In the following, DMC will refer to the Council unless otherwise stated. The special committees prepare draft assessment reports and treatment guidelines with secretariat staff. The DMC conducts the final assessment of the value of new medicines and decides whether a medicine should be recommended as standard treatment in the country’s hospitals (Medicinrådet 2025).^2^ The DMC consists of eighteen members and four observers. Most members are doctors in leading positions at hospitals across the country. However, the DMC also includes members from other backgrounds, such as health economics and clinical pharmacology (Medicinrådet 2025).^3^ The Danish regions appoint the DMC’s chairs. The remaining members are appointed by the regions, the Danish Society of Medical Sciences (LVS), Danish Patients, and the DMC itself (Medicinrådet 2025).

The main difference between the DMC and its predecessors is that the DMC takes prices (i.e., the costs of medicines) into account. However, it only does so with medicinal costs (and possibly, in the broader health economic analysis, potential savings on care/home care borne by the regions and municipalities) and not costs/gains related to productivity/labour market participation/taxes. When we conducted the study, the DMC measured treatment effects by (disease-specific) *clinical value added* (“klinisk merværdi”). This measures how much improvement a new drug provides patients compared to the current standard treatment. It is based on multiple factors, such as effectiveness, safety, and patient outcomes.^4^ Since then, the DMC has moved to using QALY (but without a QALY threshold), but their guiding values remain the same.

What values guide the DMC? Initially, seven principles were drafted: professionalism, independence, geographical equality, transparency, rapid use of new medicines, more health for the money, and access to medicines (Sundheds- og Ældreministeriet 2016). Later, two more principles were added: severity and precautionary (Medicinrådet 2020; 2024). The DMC is not in charge of purchasing or allocating economic resources for the purchase of medicines, does not work within a fixed budget, and is limited to issuing recommendations. However, since hospitals largely follow its recommendations, its decisions have far-reaching rationing consequences for health budgets throughout Denmark.

It should be noted that although the decisions taken by the council in the DMC are important for priority setting and are therefore the focus of this article, important decisions are also taken elsewhere. The special committees and their work are important for the outcome, and the Regional Medicines Committees play an important role in priority setting in Danish healthcare. The latter do so mainly because individuals (doctors) can request the use of a medicine for a specific patient even if the DMC does not generally recommend it.

As a case, the DMC is interesting both in itself and as a member of the broader group of similar rationing agencies worldwide. First, it is interesting simply because it significantly influences how large sums of money are distributed among different patient groups and how efficiently these sums are used. Knowing how different concerns inform decisions and how DMC members perceive their role is an important starting point for a critical discussion among DMC members, politicians, and the wider Danish public about the values and concerns that guide and *should* guide the allocation of healthcare resources. Second, the DMC is only one of many similar rationing agencies worldwide. These councils differ in their institutional design, and the findings in this case cannot be generalized easily to others. However, studying singular cases such as the DMC can contribute to the international learning processes already taking place.^5^

## Data and Methods

### Research Design

Our study explores how the DMC makes priority setting decisions, focusing on the concerns and values informing these decisions and how the members perceive the identified concerns and values. We employ a qualitative research design, which combines semi-structured in-depth interviews with DMC members and observations of DMC meetings. Our approach is grounded in an interpretivist perspective, aiming to capture the meanings and understandings of those involved in priority setting.

### Combining Interviews and Observations

In the spring of 2021, we conducted semi-structured interviews with eighteen of the nineteen DMC members (fourteen full members and four observers) and observed three subsequent meetings (February, March, and April). Combining these methods proved beneficial in several ways. Observing meetings was initially challenging due to unfamiliar medical terminology and implicit contextual knowledge. Conducting interviews concurrently enabled us to clarify discussions and assess the extent to which the observed meetings were typical. Additionally, using real-time cases from meetings as discussion points in interviews helped interviewees articulate their perspectives more concretely than they might with hypothetical vignettes. An important role of the meeting observations was to provide the relevant background knowledge and familiarity with how the DMC operates, to ask the most relevant questions during the interviews, and to be able to ask participants about real ongoing cases. Finally, while interviews captured subjective meanings, observations provided insight into how these concerns manifested in deliberations, helping to mitigate discrepancies between stated and actual behaviour. Observations also reduced social desirability bias as participants responded within a group context rather than solely to an interviewer.

### Interviews

We individually invited members to participate after introducing our research project at the February 2021 meeting. Due to COVID-19 and scheduling constraints, all interviews were conducted via Zoom and followed general guidelines for semi-structured interviews. Dorf conducted all the interviews. Participants provided informed consent and were briefed on the research aims and data usage. While virtual interviews may be less conducive to sensitive discussions, we considered this a minor concern, given the professional nature of the topic, especially since the interviewees had extensive experience with such forms of communication due to the pandemic.

The interviews examined how members applied principles, reflected on challenging cases, and viewed the DMC’s role. Interviewees were asked to consider rationing decisions that had made a particular impression on them. These cases served as specific discussion points, offering insights into competing concerns and aiding recall. Initial open-ended questions about the DMC’s role and key decision factors gradually transitioned to more focused queries on specific principles (e.g., the severity principle) and considerations (e.g., age). The interview guide was adjusted as needed to reflect recent meetings and interviewee remarks (see online supplementary materials). All interviews were audio-recorded and fully transcribed in Danish by a research assistant.

### Observations

The three observed meetings spanned five days. In total, we observed twenty-five hours of meetings. The meetings were held via Microsoft Teams due to COVID-19, with members participating remotely. Unlike the interviews where the virtual setting had minimal impact, the online format of meetings may have affected the deliberative dynamics of the DMC meetings. We asked interviewees whether these meetings differed from in-person sessions to assess this. They noted some differences, such as quicker consensus due to the lack of informal break-time discussions. However, the overall conclusions were not perceived as significantly affected. Council members, sub-committee members, and administrators participated in the meetings. Due to the number of participants, including other observers at the meetings, and because of their virtual nature where we, the researchers, were hidden behind anonymous screens (without sound and image), we came very close to the positivist ideal of observers as “flies on the wall.” In interpretive research, this is often less of an aspiration as the positionality and participation of the researcher is often actively used. However, even if we had attended the meetings physically, it would not have been possible for us to participate and interact with the members in an informative way, and online participation may have minimized social desirability bias, which we consider to be a virtue in our specific case.

While observing the meetings, each researcher present made condensed field notes and jottings to detail emerging themes from the discussion. These were made for each point on the agenda. After each day of observation, expanded field notes were made based on the jottings and notes during the meeting. These primarily aimed to expand on what was observed in the descriptive fieldnotes (Bernard 2017, 355; Spradley 2016, 69; Emerson et al. 2011, 45). The last task to be completed was to add analytical field notes to the end of the document (Bernard 2017, 356; Spradley 2016, 72; Emerson et al. 2011, 123). These main points were to note observed themes and conduct more analytical notes. The observation notes from each researcher present were combined into a single observation note, which was used for the analysis.

### Coding and Analysis

Once the interviews had been transcribed and the notes from the observations had been merged, we coded the data from the meetings and interviews. We coded the data in two stages. First, we conducted open coding to identify emergent themes relevant to our research question, prioritizing close adherence to the data. Second, based on the themes emerging from the open coding, we developed a fixed coding scheme of closed codes. All three authors independently coded the same two interviews to ensure reliability, comparing results to refine coding guidelines. After this, we divided the interviews and meetings between us, and Nielsen and Albertsen coded all the data from the remaining interviews and observation notes. The final coding scheme is included in the Online Supplementary Materials. All coding and analysis were conducted in Danish to maintain accuracy. Translated quotations in the analysis serve as power quotes, illustrating key findings (Pratt 2009).

### Research Ethics

The DMC was an external collaborator on the research project and approved our access to meetings. The DMC did not commission the study and has not influenced any parts of the study. The access granted came with some limitations aimed at ensuring that our study did not affect the DMC’s ability to discuss freely or compromise economic details on the part of the applying companies. This means that we are not allowed to quote or attribute views in other ways to the individuals who expressed them during a meeting. While we can refer to arguments and sentiments expressed, we cannot do so in a way that reveals which product this was said about. Additionally, we were not allowed to record the meetings. Before the interviews, the DMC members were informed about our project, the study’s purpose, and the interviews’ role in this. They were also assured that their names would not be used in the articles and that the interviews and transcripts would only be available to the researchers behind the study.

## Results

In our analysis of the meeting observations and interviews, several important themes related to our inquiry emerged. Each is important, but together, they provide a fascinating picture of what DMC members perceive to be important in their decisions. This first section describes how the lack of adequate data impedes the ability of DMC members to assess the effect of newly proposed medicines adequately. In these situations of uncertainty about effect, recommendations are influenced by considerations such as the age of patients and the rarity of the disease.

### The Primacy of Effects and the Relation between Price and Effect

One important thing that emerges from both interviews and observations is that, overall, DMC members see it as their primary task to evaluate whether a medicine has the desired effect. That is, whether it improves patient outcomes relative to available treatments. As one DMC member puts it:

I would say that you can boil it all down to, eh, first you have to decide whether a drug works at all. Does it have any effect at all, or does it do more harm than good if you put it in the patients. (“Interview 2” 2021)

DMC members have expressed this primacy of effects view in several interviews. Our observations at the meetings confirm this primacy of effect. Here, we noted how price discussions were not initiated for medicines deemed ineffective. Furthermore, much of the deliberation focused on the magnitude and certainty of effects. Discussions about price were then a secondary manner. Sometimes a clear hierarchy was expressed:

We can say that even if something is only almost as good, we can choose something cheaper, but we can’t say that something is good because it’s cheap. (“Meeting 1 Notes” 2021).

While the primacy of effect view is commonly expressed among DMC members, some interesting variations exist. One member criticized instances where price was considered despite a drug having no significant effect. Another noted that if a drug’s effect is sufficiently strong, price may become irrelevant:

I would say that price is close to not being a part of it … I don’t think you can cite a single example of a disease that is even remotely serious, where there is a drug that clearly works, and then it is not accepted. (“Interview 7” 2021).

The dominant view identified in the interviews and observed in the meetings is one in which the effects of the drug are significant. The price is important and a central part of what the DMC should include in its assessment, but for medicines that work, and where the effect is significant, price becomes a less central factor. However, this conclusion is complicated by another key feature that emerged from the interviews: there is considerable uncertainty about these effects.

### Uncertainty about Effects and Insufficient Data

A recurring challenge identified in both interviews and observations was the lack of sufficient data to assess treatment effects and side effects properly. As one DMC member noted:

Our big problem is, eh, is typically a lack of knowledge, it’s… it’s very often an uncertainty about treatment effect and then it’s an uncertainty about, eh, an uncertainty about side effects. (“Interview 1” 2021)

This lack of sufficient data was also mentioned in other interviews as a route to difficulties and eventual rejection. DMC members have widespread reservations about the quality of the data in the studies presented as evidence of the proposed drugs’ effects. This uncertainty means that many disagreements within the DMC revolve around different interpretations of the medicines’ effects based on inadequate data quality. These accounts by DMC members were clearly consistent with our observations of the meetings. At all meetings, the lack of sufficient data was an important part of the discussion in many cases. Some DMC members also explain that they see this as an increasing problem.

Thus, a recurring theme that emerged in our interviews is that instead of assessing price and effects, a core task of the DMC is to make an assessment of effects. However, this apparent willingness to pay for things that work is, of course, highly complicated by the fact that there is a great deal of uncertainty about whether things work.

While the uncertainty of effect arguably changes the nature of the price/effect trade-off, it does not make price unimportant. A clear demonstration of how the price may matter was observed at one meeting, where a medicine that had previously been rejected was now accepted. The data was the same, but the price was lower. So, a driving thought, in terms of the principles, is that there must be a reasonable relationship between cost and effect. This view was expressed in several interviews, and the sentiment was present in all the meetings observed. This is not in itself surprising (Petersen et al. 2023), but it is interesting to see how large a role uncertainty plays here.

Based on the above, it seems fair to say that the most important aspect of evaluation is effect. However, this aspect is complicated by the recurring experience that the poor quality of the available data creates considerable uncertainty about effects (and side effects). When the uncertainties mean that we do not know whether a drug will work (or work as well as the currently available drugs), the willingness to pay is reduced. This is the main trade-off considered by DMC members and observed at the meetings. If a drug is deemed to have a positive effect, there is a willingness to pay (tempered by any uncertainty)—but there needs to be a proper proportionality between the two. The uncertainty about effects is a key point because it shifts the focus and debate among DMC members away from assessing the ratio between price and effect and towards interpretations of effect based on poor data. Before discussing this result, we will briefly look at some other factors that seem to increase willingness to pay even in the face of uncertainty about the effect.

### Rarity of Disease

If a disease is rare, several DMC members see this as a reason to increase willingness to pay, even if there is considerable uncertainty about how much good the drug will do (and in line with the seventh DMC principle, which highlights the importance of access to treatment). One DMC member believes that there may be indirect affirmative action in DMC decisions regarding rare diseases. Other DMC members say in interviews that they believe there should be a higher willingness to pay for rare diseases. Some point out that it is more expensive and challenging to conduct research and development for medicines for rare diseases, and the resulting the low-quality data has to be accepted to a larger degree, while others note that decisions for rare diseases may be cheaper in terms of overall costs (because they affect few people) and therefore approved despite high costs per patient. While these sentiments are observed in several interviews, some dissenting views should also be noted. One DMC member, for example, expresses disagreement with the idea that when a patient population is small, willingness to pay should be higher. These observations suggest that despite the disagreement, there is some tendency to consider disease rarity as a relevant factor that increases willingness to pay even in the face of uncertainty of effect.

### Children and Severity

The fact that those receiving a drug are children can be an independent concern that increases willingness to pay. We see this with several DMC members. Sometimes, the fact that children are involved is treated as a moral issue in its own right. To see this, consider the following statement:

These are children, and you really really want to do it, eh, and I can easily see that the data is really bad, but it’s still about children, and you’re saying yes and no based on very poor data, and I can easily see that sometimes you have to say no because the data is so poor, but, but these are still cases where you want to do the very best, and it’s just hard to know what the very best is. (“Interview 16” 2021)

In other cases, the higher willingness to pay despite uncertainty about effect is justified by the fact that the treatment of children is a relevant part of the interpretation of the severity principle (in line with the official interpretation). It is suggested in several instances that the severity principle is used more often in cases involving children—something we also observed as a suggestion in one meeting. Other reasons for such a priority are also given. Some DMC members explain that the effect-related importance of early treatment will, in some instances, serve as a reason to give priority to drugs for children even when the effect is uncertain. Several DMC members also mention that giving higher priority to children reflects the views of the public. There are a few dissenting voices, but in general, the “children factor” results in a higher willingness to pay despite uncertainty about the effect.

### No Other Available Treatment

Another factor that is sometimes relevant is whether the proposed medicine is the only treatment available. It is emphasized that if no other treatment is available, it is particularly urgent to obtain one, and therefore, the lack of alternative drugs increases the willingness to pay. This concern came up in discussions at all the observed meetings. Although relevant, this consideration is less important than the previous ones and was seldom brought up in the interviews.

### Practicalities for the Patient

Another observed concern, and less frequently mentioned by DMC members, was the practicalities for the patient. That is, how difficult it is to administer. This came up in discussions at all the observed meetings. In the interviews, some relayed that this was particularly important in cases where more vulnerable parts of the population frequently used medicine. While this consideration came up in all the meetings, it was rarely raised as being especially important in the interviews.

## Discussion

The above provides important insights into the DMC and the priority setting practices undertaken there. We have the impression that for many DMC members, the primary value lies in the effects of the drugs under consideration (including side effects). These effects are complicated—both in the observed reality of meetings and in the experience of DMC members—due to the poor quality of data and the resulting uncertainty about effects. Increased effectiveness increases willingness to pay for them, but this is significantly moderated by the uncertainties about effectiveness. DMC members strive to achieve an appropriate relationship between price and effect, and several members consider factors such as the rarity of the disease, the lack of other treatments, and the age of the recipients to be factors that influence what is considered an acceptable relationship in terms of increasing willingness to pay.

We find that DMC members see it as a key objective to recommend new medicines based on an appropriate balance between price and effect. To this end, it is essential to make judgments about both price and effect. Still, our analysis suggests that health effect is generally more important to DMC members than price and that assessments of effect take precedence over price considerations in discussions. The conditions for assessing the effects are often associated with uncertainty due to poor data quality and limited documentation. Consequently, in many cases, the main task of the DMC shifts from simply making a recommendation based on the relevant information on price and effect to attempting to provide an estimated interpretation of the effect based on the available data and making recommendations in the light of that estimate.

Thus, the main finding of our study is that uncertainty about the effect of the proposed medicines is a key driver of the relevance of including normative considerations in the evaluation (e.g., rarity of disease, children, other alternative treatments, and practicalities), which, given the uncertainty, may have a significant impact on the DMC’s willingness to pay. This is probably because such considerations are relevant to willingness to pay when the effect of drugs is uncertain. On the contrary, when the effect of the medicine is known (i.e., significant effect or insignificant effect), willingness to pay appears to be largely unaffected by such normative considerations.

Under these conditions of data uncertainty, several external considerations influence DMC members’ willingness to pay and ultimately their recommendations, some of which raise significant ethical questions. Our study reports that the rarity of the disease is an important positive factor in DMC members’ willingness to pay. DMC members give various reasons for accepting both a higher price and more uncertainty about the effect when it comes to rare diseases. Suggested explanations include the additional costs involved in testing the effect, lower expected revenues due to a small patient population, and fairness in providing medicines to minority patients. This finding raises interesting questions for the further justification of these judgments.

In particular, the DMC’s general consensus on increased willingness to pay based on rarity of disease is highly controversial in the medical ethics literature and runs counter to the standard call for fair treatment of patients regardless of their condition. The standard argument against priority based on rarity of disease is based on considerations of fairness and non-discrimination. Intuitively, it may seem that we owe a special duty to people with rare diseases because whether a person has a rare or common disease is essentially a matter of bad luck and not something for which people can be held responsible. Therefore, our healthcare priorities should not punish people for rarity that is not their own fault. However, as medical ethicists have pointed out, since people are similarly not responsible for common, non-rare conditions, we have an equivalent moral obligation to treat them. It follows that given budget constraints and scarcity of medical resources, we are not justified in giving rarity, at least in itself, a primary role in our priority setting assessments (Albertsen 2022; Juth 2017; Juth et al. 2021; Magalhaes 2022). The consideration of disease rarity therefore calls for further moral justification.

The study also shows that there is a strong tendency to accept higher prices and more data uncertainty on effects when it comes to medicines that could potentially benefit children. Underlying this widespread trend, however, are a number of different suggested justifications that raise important normative questions. We might speculate that we have a special duty of fairness towards children with early life conditions because such patients have not yet had a fair chance at life—i.e., a “fair innings” consideration (Bognar 2015; Nielsen 2020)—which could potentially affect willingness to pay. However, while appealing, the fair innings argument is contestable, partly because it is difficult to identify the threshold for having had a fair chance at life (Bognar 2015; Williams 1997), and partly because it requires further argumentation to justify the special moral status of childhood (Gheaus 2015). Moreover, DMC members are divided on whether such age-based fairness considerations can and should be taken into account.

Giving special priority to children could sometimes be justified on the basis of health effects, as some DMC members argue. This is because the long-term effect of a given medicine is often better if it is given at an earlier stage. However, it is unclear whether the justification is based on the better prospects for effect when treatment is given at an early stage of the disease or on the overall societal benefit of treating young patients, or both. Regardless of the interpretation, however, these arguments need further normative justification and transparency, partly because, as noted above, they only become relevant when the effect is already questioned, and partly because they may involve indirect age discrimination (Lippert-Rasmussen 2013).

In conclusion, in order to fulfil their obligation, the members of the DMC are often obliged—in part due to relatively poor data quality and considerable empirical uncertainty—to carry out extensive evaluations involving a number of contestable, implicit normative assumptions and assertions for which they are not necessarily professionally trained. These practices raise difficult ethical questions and require further moral justification. This finding has important implications for how we understand the general ethical underpinnings of priority setting institutions.

These findings provide an important insight into real-world priority setting and how those who carry it out see it. It provides a description of the practice of the DMC, which gives effects and assessment of effects a larger role than cost-effectiveness judgements. This is interesting because cost-effectiveness is very often considered to be the at the very core of what the DMC is and does. A second reason why our findings are interesting is that the philosophical debate over priority setting shares this affinity for placing cost-effectiveness at the centre of the discussion without properly acknowledging the uncertainty about effects that apparently influence real-world priority setting decisions.

Our result has theoretical as well as practical implications. It sheds light on the often-overlooked uncertainty and ambiguity involved in reference to the value of cost-effectiveness. This is an important input to the bioethical literature on healthcare priority setting where cost-effectiveness is typically taken as an important and robustly defined value, centrally positioned in disputes about output and fairness. Moreover, from a practical perspective, the result underscores the importance of articulating values and making value-based judgements in healthcare priority setting, and thereby, the study reaffirms the need to combine normative reasoning and empirical evidence in healthcare decision-making (Charlton et al. 2024). The result should, thus, be of interest to medical ethicists and medical professionals as well as policy planners and decision-makers.

## Supplementary Information

Below is the link to the electronic supplementary material.

Supplementary file1 (DOCX 43.9 KB)

## Data Availability

The participants of this study did not give written consent for their data to be shared publicly, so due to the sensitive nature of the research supporting data is not available.

## Footnotes

1 In 2021, the DMC had fifty-three of such Committees, with 480 committee members (Medicinrådet 2022). A total of 480 expert committee members, distributed across fifty-three committees, form the foundation of the Council’s drug assessments with their professional and personal expertise in their respective disease areas.

2 Additionally, the Council approves the DMC’s treatment guidelines. These are also prepared by the special committees and the secretariat.

3 A complete list of members during the time our study was conducted can be found here (Medicinrådet 2022).

4 For example, some outcome measures are considered “critical,” they relate to mortality/survival, quality of life, severe symptoms, and side effects. The details for how this was calculated are explained here (Medicinrådet 2021).

5 For an example of such learning processes, see this description of the collaboration between NICE (United Kingdom) and the DMC: https://indepth.nice.org.uk/case-study-denmark/index.html
