# Supplementary material for: Priority-Setting and Values: A Qualitative Study of the Danish Medicines Council
Source: J Bioeth Inq. 2025 Oct 1;23(2):249–60. doi: 10.1007/s11673-025-10470-3 (PMC13388422; doi:10.1007/s11673-025-10470-3)
Supplement: Supplementary file 1 — (DOCX 43.9 KB) [file 11673_2025_10470_MOESM1_ESM.docx]

Dorf, A.C., A. Albertsen, and L. Nielsen. 2025. Priority-Setting and Values: A Qualitative Study of the Danish Medicines Council. *Journal of Bioethical Inquiry* 23(2).

**Supplementary Material**

**Observation Guide**

Translated to English, original in Danish.

Below is my practical suggestion on how the observations can proceed. The more profound methodological considerations/justifications (and the inclusion of more methodological literature) will follow in another document later. We will, of course, evaluate after the first observation. Please feel free to comment.

Overview

1. Preparation
2. Participants
3. Observation roles and themes
4. Field notes
5. Literature

**1. Preparation**

Preparation can include:

- Reading the sent material, familiarizing yourself with the agenda (and possibly the organization of the Medicines Council if necessary)
- Printing a list of meeting participants so you can have it in front of you and possibly the programme
- Trying out Teams
- Being clear on how to take field notes (see below)—remember to have plenty of paper and good pens
- Setting aside time after the meeting to transform jottings into (descriptive, analytical) field notes and to write a log/diary (I know it is demanding these days with limited time and children—but if you don’t write it down, you forget!)
- Knowing who we need to interview immediately after the meeting?
- Anything else?

**2. Participants**

Meeting participants: “The Council constitutes the top management of the Medicines Council. The Council consists of sixteen members and four observers. The Council makes the final assessment of the value of new medicines and decides whether the medicine should be recommended as standard treatment at the country’s hospitals. The Council also approves the Medicines Council’s treatment guidelines, which are prepared by expert committees and the secretariat. Most council members are doctors in senior positions at hospitals across the country, but the Council also includes a health economist, two clinical pharmacologists, two patient representatives, and a hospital pharmacist. The Council’s chairpersons are appointed by Danish Regions. The remaining members are appointed by the regions, the Medical Societies (LVS), Danish Patients, and the Council itself” (Medicinrådet 2021).

**3.** **Observer roles and themes**

We are three people observing the meetings.

There are two conflicting considerations that have implications for how open or focused our observations are. On the one hand, we want to avoid imposing our expectations/categories too much on what is observed—it wouldn’t hurt if we actually learned something surprising (cf. e.g., Emerson 2011, chap. 5). On the other hand, we are only in the field for a very short time, and we have some questions we want answers to—although we must be open to the possibility that these questions may prove irrelevant.

We take advantage of being three observing and divide roles (which we will evaluate after the first observation):

1. Open observation. This person should probably write down their expectations/preconceptions to possibly avoid them subtly guiding their attention (to the extent that such a thing can be avoided). My own include, among other things, an expectation that there is a lot of focus on clinical details and perhaps less on ethical dilemmas, and that the council members, by virtue of their positions and experience, are somewhat confident in their views. Open/non-thematic observation means (cf. Emerson’s chap. 5) that one can still be particularly attentive to certain “moments” that are suitable for uncovering “indigenous meanings” (the list is solely for inspiration – read it and then “forget it” again): ~ What questions do participants find relevant to ask? ~ What contrasts are drawn? ~ What words/typologies are repeated/surprising/attention-grabbing? ~ How are specific groups/events described or named? ~ What do people react to as unexpected/surprising? ~ What is surprising/unexpected to you? It may also be worth noting the atmosphere, forms of address, etc.
2. Focused/thematic observation, centered around these themes: a. VALUES: What values/principles/considerations are mentioned (e.g., necessity, effectiveness (price/value), discrimination, etc.)? b. VALUE CONFLICTS: What (value) conflicts are mentioned (if any) – are they even aware that values can conflict with each other, or are they discussed as independent dimensions? c. BALANCING: How do they, if relevant, reach a balance of values/resolve conflicts? d. CLINICAL APPLICATION OF GIVEN PRINCIPLES VS. (ETHICAL) DISCRETION: Is the language predominantly “clinical” (descriptive) or “moral”—does that categorization even make sense here? In this context: Do they speak as if they are neutrally applying top-down principles—or as if they are making independent decisions? e. PROBLEMATIZING DISCRETION/REGULATION? Is the balance between discretion/top-down guidelines mentioned/problematic (explicitly or implicitly—e.g., frustration over vagueness, etc.)? This person should probably also make their preconceptions explicit in a diary/log (see below).
3. Initially, I considered whether the third person should focus on how time is used in the meetings. I’m not sure if it’s a good idea—also given that we are only present for part of the meeting. On the other hand, we could perhaps try it at the first meeting and see if it provides any insights? This person could, for each agenda item (cf. the plan above), note how time was spent—clinical details vs. value conflict, finished presentation vs. discussion. The time intervals allocated to each item are short (fifteen minutes), so it should be possible to keep track of the time periods. Alternatively, the third person can observe in role 1 or 2. There may not be a significant difference in what is seen from the three roles—and that doesn’t really matter.

**4. Field notes**

I will distinguish between different types of field notes below (mainly based on distinctions in Emerson (2011), Spradley (2016), and Russel (2006)):

1. Jottings/condensed field notes (e.g., Emerson: 29ff, Spradley: 63ff, 349f): These are notes quickly jotted down in the field (i.e., during the actual observation). I clearly believe it is preferable to write these by hand – it needs to be quick! To end up with data of the highest possible authenticity (where it becomes easy for the recipient to follow the path from observation to interpretation), the following is important: ~ Write as concretely as possible. For example, not: “the children fought for my attention on the way to the park” but instead “A pulled my arm, B kept interrupting, C spoke increasingly louder.” This can make it possible to show how the later interpretation is anchored in the observation of behavior. Be careful not to summarize/generalize/use stereotypes: “the mood was generally” … Write the observations that give rise to that interpretation, show it, don’t tell it! Details! ~ Try to write as verbatim as possible. If possible, include whole quotes. Preferably direct speech like: CBH: “The effect of the medicine is not good enough compared to”. ~ In continuation of this: Write in the language the participants themselves use—avoid, as far as possible, analytical/theoretical terms (that they do not use themselves) at this stage.
2. Expanded: These notes are taken after the observation and can be divided into two types:

a. Descriptive field notes (Russel: 355, Spradley 69 ff, Emerson: 45 ff) Descriptive field notes are written (immediately!) after the observation and expand on the jottings—the advice regarding jottings above (write concretely, write verbatim, etc.) also applies here. These notes can be structured in various ways—I suggest we initially structure them chronologically according to the meeting points. The descriptive field notes can include both what happened, etc., but also provide a grand tour of the scene, most relevant for us: who was present, how did they appear, how many were there, etc. (According to Emerson and Spradley, even descriptive field notes are, of course, interpretive—you have noticed certain things and ignored others. But you keep the interpretation to a minimum.) You can consider whether to write in the first person or third person (cf. e.g., Emerson chap. 4)—since we are not so immersed (we are more observers than participants), I suggest third person as a general rule.

b. Analytical field notes (Russel: 356, Spradley: 72, Emerson: 123) The analytical notes are also started immediately after the observation (but after the descriptive ones, I suggest). The analytical field notes take on a more interpretive character – this is where the analysis begins. What does the observed mean? What themes are interesting? What patterns emerged? What language was spoken? What are the contrasts, etc.? See, for example, Spradley Step 5 (p. 84) (though somewhat banal and at the same time somewhat cumbersome).

c. Methodological field notes (Russel: 354) Some prefer to have these together with the other field notes —in our case, I prefer to place them in the log. What worked well methodologically/what did not—what should we change, etc.?

1. Log (e.g., Russel: 351): Here you can write the plan for the observation—and subsequently, how it actually went. It is not as important in our case (only one location, and we are quite sure the meeting will take place)—whether you find it useful, I think, is up to you?
2. Diary (e.g., Spradley: 71, Russel: 350): Again, perhaps not so relevant in our case, where the stay in the field is short.

We can possibly use the following schema for the field notes (feel free to suggest changes). Jottings are probably first taken by hand but should subsequently be written down. If you agree, I will later put it into a separate document.

| **Agenda point** | **Jottings** | **Descriptive notes** | **Analytical notes** |
| --- | --- | --- | --- |
| **9** |  |  |  |
| **10** |  |  |  |
| **Pause** |  |  |  |
| **11** |  |  |  |
| **12** |  |  |  |
| **14** |  |  |  |
| **15** |  |  |  |

**5. Literature**

*Webpages*

Medicinrådet 2021a: Rådet. https://medicinraadet.dk/om-os/organisation/radet.

Medicinrådet 2021b: Sekretariatet. https://medicinraadet.dk/om-os/organisation/sekretariatet.

*Books*

Emerson, R.M., R.I. Fretz, and L.L. Shaw. 2011. *Writing ethnographic fieldnotes*. Chicago: The University of Chicago Press.

Russell, B.H. 2006. *Research methods in anthropology. Qualitative and quantitative approaches*. Lanham: Rowham.

Spradley, J.P. 2016. *Participant observation*. Long Grove, IL: Waveland Press.

# Focused Coding List

Translated to English, original in Danish.

The code list reflects the two main themes we ended up focusing on:

1. The considerations (principles, values, etc.) that form the basis for prioritization decisions, which considerations are important/less important, and how these considerations are understood/interpreted/put into play.
2. The relationship between these considerations (harmony/conflict, do some considerations have lexical priority/do some have the status of “sacred values” that cannot be “traded off” against “profane values”) and how to handle it if the considerations are in conflict.

We decided to exclude the role/self-perception of the Medicines Council in this article except where it is relevant to the above.

Because the considerations are predominantly based on the open codes and therefore reflect the way the members of the Medicines Council think about the considerations, it cannot be expected that the code list follows a logical/theoretical structure. The goal is not to rationalize the considerations but to describe which considerations actually come into play and how. The codes are therefore not mutually exclusive – there will often be overlap.

I have created three levels of codes, as also shown in the table below:

- Considerations
  - Codes
    - Subcodes
- Relationships between considerations
  - Codes
- Notable cases
  - Codes

Since there is only a third level under considerations, it may be easier to handle in NVivo if you drop the top level (considerations) and just code a text segment with, for example, “cost-effectiveness” ˃ “price” instead of “considerations” ˃ “cost-effectiveness” ˃ “price”. You will have to assess that.

I suggest that we generally code relatively long sequences (several sentences, up to half pages where necessary) rather than completely isolated sentences or words. Much of the meaning comes from the context, and it is easier to cut something out in displays/analysis than to guess the meaning of a sentence because the context is missing. This does not mean that you should always code long sequences—sometimes only short ones are relevant.

| **Codes** | **Subcodes** | **Description** |
| --- | --- | --- |
| **1 Considerations** | | |
| **7 official principles** |  | The 7 official principles when mentioned as a whole package. There will sometimes be overlap with the separate codes for each principle |
| **Cost-efficiency** |  | Cost-effectiveness refers to the price/cost for a given effect (excluding side effects) according to the 6th principle “more health for the money.” |
|  | Health effect | Treatment effect includes clinical added value, disease-specific effects such as survival, improvement in functions, etc., and quality of life. |
|  | Side effects | Side effects of the treatment |
|  | Price/cost | Price of the treatment. If the consideration is about the overall impact on the budgets of regions/hospitals, it is not included here—it is included under the precautionary principle. |
| **Principle of precaution** |  | For example, discussions about it not being dangerous to recommend even if it is expensive per patient because the patient group is so small that it has minimal economic consequences overall. For example, considerations about whether it can be argued to recommend to a smaller group (if it appears that the budget impact is the real motivation) |
| **Equal treatment** |  | Under this code, statements concerning (i.e., both for and against) discrimination, equal treatment, etc., are included. |
|  | Age | Statements about equal treatment or discrimination based on age. For example, statements saying “there is a special consideration for children” or statements saying that age discrimination is wrong. |
|  | Disability | Statements about equal treatment or discrimination based on disability (or possibly characteristics associated with disability, such as quality of life or life expectancy). |
|  | Ability to work | Statements about equal treatment or discrimination based on the ability to maintain or regain work capacity or the ability to contribute to society/tax revenue. For example, statements about a medicine “paying for itself” (because the patient can work and pay taxes) and arguments for or against including broader societal costs/benefits |
|  | Responsibility | Statements about equal treatment or discrimination based on responsibility for the disease. For example, statements about responsibility for smoking, obesity, etc. |
|  | Vulnerability | Statements about equal treatment or discrimination based on social vulnerability/special vulnerable groups/ability to comply. |
|  | Rarity | Statements about equal treatment or discrimination based on the rarity of the disease (small patient groups) (cf. also the 7th of the official principles, which gives patients with rare diseases equal access to treatment) |
|  | Specific conditions | Statements about equal treatment or discrimination based on specific diseases (e.g., cancer, diseases with strong patient groups) |
|  | Severity | Statements about equal treatment or discrimination based on the severity of the disease. Note that severity in the official note (here) covers more than it usually does in the literature (e.g., a disease is considered more severe in these guidelines if it targets children or if there is no other available treatment—not only if it is very deadly/burdensome), and that the members of the Medicines Council are not always entirely clear on what it covers. Severity principles are rarely explicitly the basis for decisions, but that does not exclude the possibility that severity as a consideration is included more often |
| **“Leave none behind”** |  | Empathetic consideration for the individual patient. All patients should have a chance. Uncertainty should benefit the patient. Special priority if there are no other available treatments. Note: I have made this a separate code (rather than, for example, including it under equal treatment) because I think the phenomenology is different—it is in situations where the medical empathy for the specific patient somehow shines through. I don’t think it will be used often—it shouldn’t cause major problems |
| **Practicalities for patients** |  | Consideration for the practical burden on patients. Does NOT include side effects of the medicine. For example, the practical burden of having to show up at the clinic/take a daily pill or similar |
| **Practicalities for health system** |  | Consideration for practicality/feasibility regarding implementation in the healthcare system. For example, can the hospitals manage it? |
| **Advice to practitioners** |  | Consideration for resolving ethical conflicts for those further down the system, so clinicians do not have to deal with it/make the decision when facing the specific patient with the ethical pressure it entails. |
| **Against off-label** |  | Consideration for limiting the use of off-label treatment. |
| **Incentives from companies** |  | Consideration for the incentives created by the Medicines Council’s decisions for companies (“dynamic effects”) |
|  | Lower price | When considering that non-recommendation can lead to a lower price in the future (“bargaining”) |
|  | Better evidence | When considering that non-recommendation can lead to better evidence/data quality/studies, etc., in the future, i.e., that companies make more effort to provide a proper evidence base. Note: Different from the independent consideration of evidence quality below |
| **Public opinion** |  | Consideration for public opinion/public sentiment/in line with societal norms |
| **Quality of evidence** |  | Evidence quality/safety/data quality. For example, when poor evidence argues against approving a drug (or accepting a high price) because one cannot be sure of the effect. |
| **Procedural considerations** |  |  |
|  | Consensus |  |
|  | Consistency | Consistency/uniformity between decisions over time |
|  | Precedence | Precedent-setting effect |
|  | Transparency | Transparency/understandable language/good justification to the public |
|  | Professionalism |  |
|  | Arm’s length | Arm’s length from politicians |
|  | Relation to other countries |  |
|  | Representation | Inclusion of relevant interests, including patient representatives |
| **2. Relationship between considerations** | | |
| **Relationship between considerations** |  | Under this code, passages concerning, for example, conflicts between considerations (or harmony for that matter) or hierarchies between considerations or similar are coded. For example, professionalism comes before price. For example, conflict between high price/low evidence and severity/age |
| **Dealing with value conflicts** |  | Under this code, passages that say something about how decisions are made in light of conflicts between considerations are coded. For example, that it is done based on gut feeling or that it is difficult to justify the decision if one consideration argues for and another against and it is unclear how they should be weighed |
